# Remarkable tumor shrinkage in hilar biliary cholangiocarcinoma confirmed by peroral cholangioscopy following neoadjuvant chemotherapy

**DOI:** 10.1055/a-2234-4355

**Published:** 2024-01-30

**Authors:** Ko Tomishima, Akinori Suzuki, Koichi Ito, Shigeto Ishii, Toshio Fujisawa, Yuki Fukumura, Hiroyuki Isayama

**Affiliations:** 1 Department of Gastroenterology, Graduate School of Medicine, Juntendo University, Tokyo, Japan; 2 Department of Human Pathology, Juntendo University School of Medicine, Tokyo, Japan

Neoadjuvant chemotherapy is considered an effective strategy for patients with advanced biliary tract cancer for improving R0 resection and prognosis 1 . We report a complete pathological response with remarkable biliary tumor shrinkage on peroral cholangioscopy (POCS) both before and after neoadjuvant chemotherapy.

A 73-year-old man underwent POCS (SpyGlass DS; Boston Scientific, Marlborough, Massachusetts, USA) with an initial endoscopic retrograde cholangiopancreatography for suspected biliary tract cancer. POCS revealed an irregular circumferential granular stricture with erythematous mucosa and ready oozing within the common hepatic duct, extending from the bifurcation of the right and left hepatic ducts to the cystic duct ( Fig. 1 **a** , Video 1 ). The left intrahepatic bile duct and anterior segment were normal; however, the root of B4 and the posterior segment had a persistent, irregular granular stricture with erythematous mucosa from the hilar region, indicative of tumor invasion. Biopsies of the right and left hepatic duct bifurcation showed adenocarcinoma ( Fig. 2 ).

**Fig. 1:**
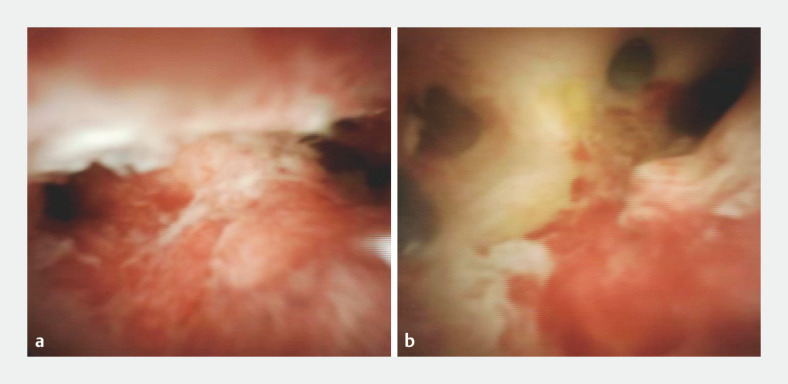
Peroral cholangioscopy findings before ( **a** ) and after ( **b** ) neoadjuvant chemotherapy. **a** An irregular circumferential granular stricture with erythematous mucosa and ready oozing were seen at the bifurcation. **b** The irregularly granulated stricture and redness at the bifurcation improved, and white bile ducts were seen.

Peroral cholangioscopy findings following eight cycles of gemcitabine and cisplatin plus S-1 therapy revealed delineated whitened bile ducts, marked by improvement in the granulated stricture and redness.Video 1

**Fig. 2:**
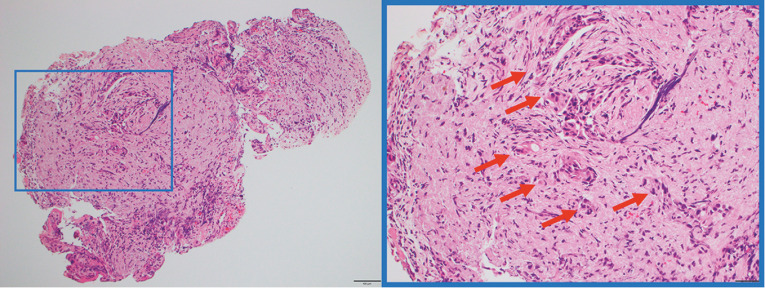
Before neoadjuvant chemotherapy, a biopsy of the bile duct at the right and left hepatic duct bifurcation showed invasive adenocarcinoma with desmoplastic reaction (arrows).

Subsequent POCS findings following eight cycles of gemcitabine and cisplatin plus S-1 revealed delineated whitened bile ducts, marked by improvement in the irregularly granulated stricture and redness at the bifurcation ( Fig. 1 **b** , Video 1 ). The erythematous tone of the common hepatic duct had also improved, with concomitant fibrosis. A section of the common hepatic duct showed a raised lesion unilaterally ( Video 1 ; arrow) and thick tortuous vessels ( Video 1 ; arrowhead), suggestive of tumor invasion; however, a biopsy showed fibrous scar formation, indicating a chemotherapeutic effect on the tumor ( Fig. 3 ).

**Fig. 3:**
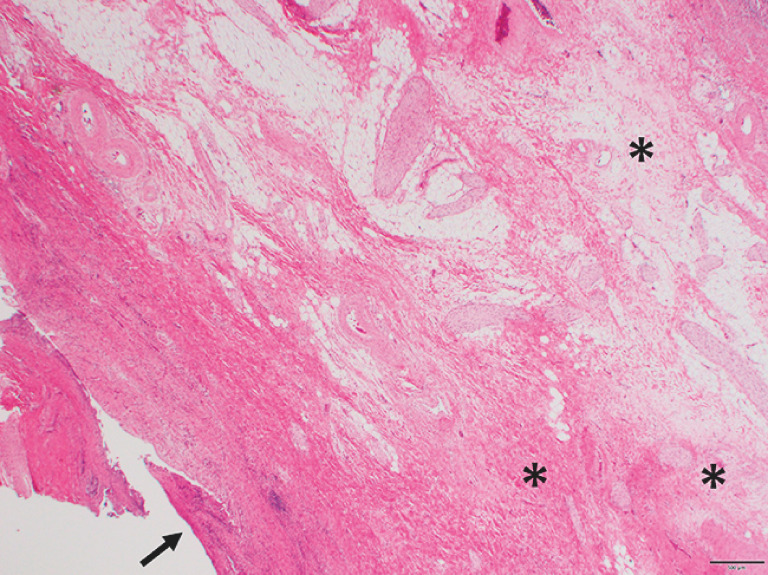
After neoadjuvant chemotherapy, a biopsy of the bile duct at the right and left hepatic duct bifurcation showed fibrous scar formation with no viable carcinoma cells (*). No epithelial cells remained on the luminal side (arrow).

An extended right hepatectomy was performed with R0 resection (stage 0, pTisN0M0). Subsequent histopathological analyses confirmed Evans’s grade IV findings, indicating that no viable tumor cells were present 2 .

The sensitivity and specificity of POCS as a diagnostic technique are reported to be 94.7% and 92.6%, respectively 3 . However, the evaluation of progression after neoadjuvant chemotherapy is difficult and requires careful judgment based on the POCS diagnosis.

Endoscopy_UCTN_Code_CCL_1AZ_2AC

## Notes

Endoscopy E-Videos https://eref.thieme.de/e-videos
*E-Videos* is an open access online section of the journal *Endoscopy* , reporting on interesting cases and new techniques in gastroenterological endoscopy. All papers include a high-quality video and are published with a Creative Commons CC-BY license. Endoscopy E-Videos qualify for HINARI discounts and waivers and eligibility is automatically checked during the submission process. We grant 100% waivers to articles whose corresponding authors are based in Group A countries and 50% waivers to those who are based in Group B countries as classified by Research4Life (see: https://www.research4life.org/access/eligibility/ ). This section has its own submission website at https://mc.manuscriptcentral.com/e-videos .
